# Professional Segmentation as a Barrier to Reform in Turkish Nursing

**DOI:** 10.1111/nin.70156

**Published:** 2026-08-02

**Authors:** Zuleyha Inceoz, David Hughes

**Affiliations:** ^1^ Swansea University School of Health & Social Care Swansea UK; ^2^ Department of Nursing Carroll College Helena Montana USA

**Keywords:** civil service culture, medical dominance, nurse management, professional segmentation, professionalisation, Turkey, Türkiye

## Abstract

This paper argues that segmentation within Turkish nursing is a major factor holding back the modernisation of nurse management. Since the millennium Türkiye's healthcare system has been transformed by NPM‐style reforms that changed financing mechanisms and strengthened aspects of performance management, but had only limited impact on nursing management. The entrenched power of the medical profession and persisting influence of a civil service ‘officer culture’ have been major obstacles to change, but a third factor is division within the nursing workforce. Our qualitative study identifies four orientations among hospital nurses. Plans for innovation proposed by bureaucratic modernisers and professional modernisers are resisted by traditionalists and pragmatists, and modernisers themselves disagree about the way forward. Western discourses concerning nurse professionalisation and leadership have had uneven impact. Interdisciplinary working is largely absent, as are the hybrid management/clinical roles and movement of nurses into executive management positions. Instead, nurse managers seek to advance a curtailed form of professional development centring of continuing professional education, evidence‐based practice, guideline development and creating spaces to exercise limited autonomy. Given the huge political difficulties of reducing medical power and achieving civil service reform, the best prospect of progress is to address the tensions within nursing itself.

## 1 Introduction

Reform of nursing roles and management structures has accelerated in several Anglophone and Scandinavian countries, driven by the consolidation of a distinctive nursing knowledge base, growing recognition of the value of interdisciplinary collaboration and the movement of nurses into general or hybrid management positions. These developments have typically followed broader new public management (NPM) reforms that shifted power from senior doctors to executive managers. Although NPM has influenced health system restructuring across many middle‐ and lower‐income countries, such countries have been slower to adopt the forms of nursing professionalisation and managerial expansion seen in the West. Among the many reasons for this are the continuing power of the medical profession in health systems organised on traditional patriarchal lines, and public‐sector employment regimes that restrict bottom‐up innovation. Yet an additional, less visible factor is the presence of internal divisions within the nursing workforce, which can lead some groups to embrace managerial or professionalising reforms while others resist them.

This article examines these dynamics through a qualitative interview study of Turkish hospital nurses, analysing how different segments of the workforce aligned themselves for or against the professionalisation agenda promoted by senior nurse managers. Turkish nursing has long been influenced by Western discourses of professional development, but has incorporated them selectively (Inceoz 2024). In several Anglophone countries, nurses have sought to build nursing theory and strengthen continuing professional education and reflective learning (May and Fleming 1997; Risjord 2011), as well as establishing nursing's role in clinical guideline and protocol development (Meerabeau 1998; Traynor 2009), and Turkish nurses have been influenced by these developments. However, the forms of interdisciplinary team working described in the international literature (Nancarrow et al. 2013; Smith et al. 2018)—particularly those that challenge doctors' exclusive authority over diagnosis and treatment—remain largely absent. In countries such as the United Kingdom, recognition of nursing's centrality to collaborative care has led to nurses' entry into management processes, the promotion of ‘distributed leadership’ (Fitzgerald et al. 2013) and the emergence of hybrid clinical–managerial roles (Spyridonidis and Currie 2016), but we found no evidence of comparable developments in Türkiye, either in published research or in our fieldwork.

### 1.1 The Impact of NPM Reforms on Turkish Nursing

Türkiye has a mixed public/private healthcare system, with around 80% of hospital beds in the public sector. Approximately a third of the healthcare workforce, including physicians and nurses working in public hospitals, are civil servants under the Ministry of Health. In the past, the healthcare financing system was fragmented, built around several separate insurance funds, each owned and run by different state institutions. However, this changed with the introduction of NPM‐inspired reforms in the early 2000s (Agartan and Kuhlmann 2019; Inceoz 2024 1). The Health Transformation Programme (HTP), implemented from 2003 onwards, created a purchaser–provider split, consolidated health insurance schemes into a single General Health Insurance Scheme and expanded affordable healthcare coverage (Polin et al. 2022). It also restructured public hospitals, established more than 20 large public–private partnership ‘city hospitals’ and introduced efficiency measures such as short‐term managerial contracts and performance‐related supplementary pay.

These reforms altered some aspects of nursing while leaving others largely intact. All‐graduate entry to nursing was introduced in 2007, followed by rapid expansion of postgraduate programmes. At the same time, Ministry of Health hospitals replaced nursing directorates with broader healthcare services directorates, a move critics argued weakened nursing line‐management structures. In contrast, the medical profession adapted to the reforms without significant loss of authority. After a brief period when non‐medical executive hospital managers were appointed, policy reverted to medically qualified hospital directors. A move to short‐term contracts for senior medical managers was counter‐balanced by the introduction of a performance‐related supplementary payment system (PRSPS), which benefitted the doctors more than other staff groups (Inceoz 2024).

Hospital nurses needed to adjust to reforms intended to modernise state bureaucracy and management structures, but also found themselves engaging with changing discourses concerning professionalism and nursing management communicated through the global nursing literature and transnational professional networks. Modernisation was therefore a multi‐faceted process involving overlapping and sometimes contradictory strands, including changed line‐management structures, efficiency and cost control programmes, audit, professional education, evidence‐based practice and guideline or protocol development. These different strands helped shape divergent orientations among nursing modernisers that are examined in this paper.

### 1.2 Segmentation and the Wider Study

A companion paper identified two structural barriers that nursing modernisers viewed as obstructing reform: the persistence of medical control over hospital work and a civil service culture that discourages bottom‐up innovation (Inceoz and Hughes 2026). Against this backdrop, the present article foregrounds a third and often overlooked barrier to nursing reform in Türkiye: the internal segmentation of the nursing workforce. Unlike structural constraints rooted in medical dominance or civil‐service employment cultures, these intra‐professional divisions are potentially more malleable and thus represent a critical—yet under‐examined—lever for change. By analysing how different groups of nurses position themselves in relation to professionalisation initiatives, the article illuminates the micro‐politics that shape reform efforts from within the profession itself.

## 2 Methods

The findings reported here come from 40 qualitative interviews with nurse managers and staff nurses in five Istanbul hospitals. The respondents were spread between two MoH public hospitals, two university hospitals and a public–private partnership ‘city’ hospital, reflecting the main public hospital types in Türkiye. The private hospital sector is slanted partly towards medical tourism and was excluded from the study. Ethical approval for the research was granted by the research ethics committees of universities in the United Kingdom and Türkiye. Participant consent was recorded in writing on a consent form sent electronically to the lead author. The Executive Nursing Association (*Yönetici Hemşireler Derneği*), a national professional body, agreed to support the study and provided a list of potential respondents in the Istanbul area, which had been selected as the research site to make the study manageable with the limited resources available.

The study participants comprise 20 staff nurses and 20 nurse managers, 12 of whom were senior managers (health services or nursing directorate managers or deputies). The research utilised a snowball sample, assembled with purposive selection of cases. The Executive Nursing Association provided a list of potential nurse respondents. Those who consented were interviewed and asked to suggest names of other potential recruits. These were contacted to assess their willingness to participate, and sifted to achieve a spread of grades, respondent characteristics and hospitals. The inclusion criteria were that participants must possess at least a 4‐year university nursing degree and a minimum of 5 years hospital work experience. Selections were made to achieve an even split of managers and frontline nurses, and to include a spread of ages and some males (a small minority in Istanbul hospitals). The research team left open the possibility of adding additional interviews if 40 interviews proved to be insufficient to achieve data saturation, but found that this sample provided sufficient rich data to end data collection.

Our sample, based on experienced nurses with bachelor's degrees, was based on our assessment that this was the group likely to be most involved in ongoing changes in nursing management. However, this introduced limitations when our attention turned to the issue of professional segmentation that emerged from nurses' interview accounts. In particular, two orientations more resistant to professional development—what we term the traditionalists and pragmatically oriented ‘new nurses’—were under‐represented. As will be explained later, traditionalism here is about affinity with two aspects of the old system: mode of entry to nursing and valuation of civil service status. Since 2007 all new entrants recruited as registered nurses must possess a 4‐year bachelor's degree, and the proportion of nurses with vocational college backgrounds is small and diminishing. Nurses without a bachelor's degree employed prior to 2007 could remain in post, but cannot be promoted above the grade of charge nurse. Three of six charge nurses in our study (C1, C3 and C6) with vocational college backgrounds had completed Open University degrees later in their careers. The only other participant taking a similar route was a middle‐grade supervisor (S3). However, a traditionalist orientation is not determined solely by mode of entry, and some 4‐year degree graduates too expressed support for aspects of the civil service system such as patronage and career security.

Semi‐structured interviews were completed between September 2021 and February 2022. Because the ongoing COVID‐19 pandemic made face‐to‐face meetings and travel infeasible, the Zoom application was used for interviews. These typically lasted between 60 and 90 minutes and were recorded with the respondent's permission using the facility within the Zoom application. The semi‐structured interviews generally followed a pre‐determined sequence of questions from a prepared interview guide, but allowed flexibility to add additional questions. The present paper focuses mainly on what respondents said about inter‐professional relations, what staff nurses and charge nurses said regarded their managers and what managers said about hospital nurses. Although the methods literature suggests that internet interviews can be problematic (O'Connor and Madge 2008; Janghorban et al. 2014), our experience was that they yielded rich data with few problems.

The recorded interviews were fully transcribed and subjected to thematic analysis. While the study was influenced by Glaser and Strauss's (1967) method of constant comparative analysis, the analysis focused more on the identification and examination of key concepts and themes than on grounded theory building as such. Overall, the approach taken was not dissimilar to the generic form of thematic analysis recommended by Braun and Clarke (2006), except that it placed more emphasis on a continuing process of comparing, coding and re‐coding fragments of data. Major themes and subthemes were identified and refined. The research team had not considered past literature on divisions or segmentation within nursing when the interview guide was developed, so much of the content of the present paper emerged from what respondents said when interviews moved away from the prepared questions. All passages identified for potential use were then translated from Turkish to English by the authors. More detail about the methods utilised can be found in Inceoz (2024).

## 3 Divisions Within Hospital Nursing

Theoretical work in the sociology of professions has long emphasised that occupational groups are not internally homogeneous but are instead characterised by segmentation, negotiated order and differentiated identities. Within hospital settings, social scientists have repeatedly shown that health professions are structured by internal divisions, yet recent scholarship has focused more on the differentiation of medical specialties (Hewett et al. 2015; Molleman and Rink 2015; Olsson et al. 2019; Bakewell 2025) than on the internal organisation of nursing. Analyses of divisions within mainstream nursing are more prominent in an earlier literature (Habenstein and Christ 1955; Carpenter 1977; Melia 1987; Dent and Burtney 1997), which remains theoretically important because it highlights how occupational groups evolve through internal differentiation rather than through unified collective action.

A foundational contribution to this perspective is Bucher and Stelling's (1969, 7) argument—rooted in negotiated order theory—that professional organisations are shaped by ‘continually unfolding internal differentiation or segmentalization (…) which arises out of differences in professional interest and professional identity’. This view positions professions not as monolithic entities but as arenas of ongoing negotiation, where subgroups pursue distinct projects, identities and strategies. Building on this insight, later research has examined how new roles emerge at the boundaries of nursing, how occupational jurisdictions are negotiated, and how identities shift in response to organisational change. Recent examples include studies of hybrid managerial–professional roles (Spyridonidis and Currie 2016; van Schothorst‐van Roekel et al. 2020), advanced or expanded nursing roles (Maclellan et al. 2016; De Rosis et al. 2023) and the development of nursing associates (Thurgate and Griggs 2023). These studies collectively demonstrate that professional segmentation is both a product of, and a response to, organisational and policy reforms.

The present paper extends this theoretical tradition by returning attention to divisions within the core nursing workforce. Consistent with Morgan's (1981) argument that professional differentiation often arises endogenously from reform processes, our findings show that internal divisions can also act as barriers to the implementation of those same reforms (Carpenter 1977; Blomgren 2003; Persson et al. 2021). The different orientations observed among Turkish nurses are best conceptualised as reciprocal typifications—shared interpretive categories through which nurses classify colleagues perceived as similar or different in terms of working practices, values and professional aspirations (Dingwall 1977, 373). These typifications align closely with negotiated order theory: they are situationally negotiated orientations rather than fixed nursing ‘types’ but nevertheless structure interactions and influence how reforms are interpreted and enacted.

Four such ideal type orientations—traditionalists, pragmatists, bureaucratic modernisers and professional modernisers—emerged from the accounts of nurse managers and staff nurses. Staff nurses often articulated these distinctions in relation to their managers, while managers used similar typifications to describe the nurses they supervised. It is important not to exaggerate the internal coherence and stability of these orientations. Several participants expressed contradictory or overlapping views, and at times some changed their standpoint depending on the issue being discussed. However, these fluid typifications reveal how internal segmentation operates as a micro‐political process, shaping the capacity of the nursing workforce to respond collectively to organisational change. In this way, the analysis contributes to a broader theoretical understanding of how reforms advance or stall not only because of external structural constraints but also because of internal differentiation and contested identities within the profession itself.

### 3.1 Traditionalists

Traditionalists is our gloss for the nurses who came in for most criticism in our interviews. Many frontline nurses expressed negative views about middle managers whom they saw as wedded to the established order, especially those who were seen to have been promoted via patronage or civil service time serving. Doctors and nurses working in Turkish hospitals are civil servants, subject to the regulations applying to government service and many believe that this stifles innovation and health improvement. The Turkish civil service culture has long been criticised for the persistence of patronage, clientism, timeserving and promotion based on reciprocal ties (Acar and Özgür 2004). Problems have been amplified in recent years by the increasing politisation of the bureaucracy and inter‐elite power struggles (Sayarı 2011). Many Istanbul nurses were members of Memur‐Sen, a trade union which had close ties with the governing AKP party. Several respondents complained that promotion often depended on union affiliation.

Appointments are purely something done by managers, regardless of merit; they may be influenced by things like the union or political views.

(N12‐F‐MoH Hospital‐PhD‐YE13)

The current health services manager is like that [sees innovation as a ‘threat’] and the manager before her was like that, too. (…) So, who offers or chooses these people? Usually, the union offers the job.

(N19‐F‐MoH Hospital‐MSN‐YE20) 2

Civil service status is associated with an ‘officer mentality’ linked to bureaucratic hierarchy, regulations, disciplinary procedures and the employment protections that civil servants enjoy. It involves personalised forms of patronage and nepotism in which managers develop special relationships with certain subordinates. Participant S3 who had worked in hospital nursing for 36 years, and now oversaw night shifts as a ‘supervisor’, suggested that these arrangements could be defended because they aided organisational functioning.

…a bureaucratic management style is preferred in the hospital in general. If nurses have to reach the head nurse (…) to reach the nursing services directorate (…) there is no direct access. What is there is an appointment system, but they can only be reached if there are personal relationships. Nepotism does not exist just for them; it exists all over the world. As long as it is fair, I'd say that nepotism can exist and can be justified, because everyone can be protected… protected by someone in some way. And if you do not protect anyone, sometimes you are not being fair. Being egalitarian does not mean being just.

(S3‐F‐University Hospital‐PhD‐YE36)

Yet as S3 went on to acknowledge, offering protection can create dependency and subservience to superiors.

There is also something called a love of armchairs, I guess those sitting in that chair do whatever the people above say in order not to lose that seat.

(S3‐F‐University Hospital‐PhD‐YE36)

Even a few nurse managers who had entered nursing with a 4‐year degree said that the bureaucratic order brought advantages in terms of predictable procedures and manageable workloads.

I don't have any trouble with bureaucracy. It is good. When I do something, bureaucracy ‐ in other words, laws and regulations ‐ makes my job easier. (…). I may have a problem as an administrator, but the ministry is quite dynamic in this regard and the problems are resolved immediately.

(M1‐F‐MoH Hospital‐MSN‐YE21)

Critics of the officer culture point to the way rules regulate many aspects of job performance, managers' ability to discipline and security of occupational tenure. Civil service regulations are said to stifle bottom‐up innovation, and come into conflict with ideas about quality improvement and evidence‐based practice that are central to the modernising discourses of the professionalisers.

The civil service rules restrict you. Lack of audit. Job security means employees do not work. In these ways, the job of managers is made difficult. So this comes with the officer mentality. You know, there's no fear of being kicked out or replaced. I have a PhD. We have a theoretical education.

(C4 ‐F‐City Hospital‐PhD‐YE24)

Nurse managers committed to professional development argue that this culture has strong negative effects on everyday patient care and communication between colleagues, hindering the team‐working they wish to encourage.

The civil servant regulations prevent good communication, because everyone has a job guarantee, they don't have to answer to anyone, they don't have to get along. Even after a fight, she still works as a nurse. No fear of losing her job. (…) Think about their mentality! How is it possible to talk to them? I say, ‘you need to give good care to the patient’. They answer abruptly by saying, ‘Should I clean up the patient? This is the job of the junior doctor, I don't care!’

(DM6‐F‐University Hospital‐BSN‐YE14)

Some respondents suggested that there are nurse managers who pay lip service to modernisation but covertly support the old culture. They claim that there is a mismatch between official statements about recruitment and promotion based on educational attainment, management training, capability, specialism or other meritocratic criteria, and the reality of advancement based on favouritism and personal characteristics. This leads disaffected modernisers to launch into a discourse about the personal characteristics of traditionalist managers.

There is a concept called the dark leader. Maybe you've met it too. For example, I really don't think that having a narcissistic personality with a bad outlook, a pessimistic character, is a good way of governing.

(N8‐F‐City Hospital‐MSN‐YE5)

Our charge nurse is obsequious, cowardly and has an inferiority complex. She is scared of the nursing services manager and head physician, and she panics when she sees them. The nursing services manager is very bad with low‐level managers and employees. She is too autocratic, gives orders. Everybody is afraid to talk to her (…) Both managers prefer old generation autocratic, harsh, traditional management.

(N17‐M‐University Hospital‐BSN‐YE6)

The professional modernisers say that traditionalists in positions of authority impede continuing professional education and try to stop the growing cohort of nurses with higher degrees from moving into management positions.

Our charge nurse [a high school graduate] is disturbed by the nurses who have a master's degree and creates obstacles for nurses who want to go on courses. When the charge nurse goes on vacation, she replaces herself with a (…) technician. She does not give power to nurses whose educational level is higher than hers because she sees them as a threat.

(N10‐F‐MoH Hospital‐MSN‐5)

At university hospitals to be appointed as a manager or charge nurse, they look at how many years you've spent on the ward. Are you a specialised nurse in your ward? I had a difficult situation in terms of getting into a managerial position. The ward manager they wanted to appoint was a high school graduate – the health services manager likes her – and I was a master's degree graduate.

(DM5‐F‐University Hospital‐BSN‐YE16)

Such comments might be construed as criticism of an older generation by a younger one, and a mindset that will be encountered less frequently as older nurses retire. However, traditionalism is deeply embedded in the civil service, an institution that is highly resistant to change and is supported by many who entered nursing via the current university education route for reasons such as career security. The civil service culture that extends down into the hospitals continues to reinforce norms of hierarchy and deference to superiors that reflect the authoritarian and patriarchal character of Turkish state structures (Kocabıçak 2023). Nursing, as a highly gendered profession associated with caring and emotional labour, finds itself confined mainly to the lower rungs of the civil service hierarchy, so that the traditionalist orientation—more than the other orientations identified—leaves female nurse managers with relatively little power.

From the other side, informants expressing traditional views say that many graduate nurses lack the basic care skills that come from long hands‐on experience of ward nursing work.

When you look at university graduates, they don't want to touch the sick. It's a constant shyness, a constant state of fear. That is, they are afraid of the patient. (…) The link there is broken. (…) If the base is missing you cannot build anything on top. So, these people will be managing services and hospitals. Management becomes a problem as well.

(C3‐M‐University Hospital‐MSN‐YE12)

Such passages from the interviews echo comments made by traditionalist critics of the UK's Project 2000 reforms in the 1990s, who complained that university‐educated nurses were ‘too posh to wash’. In Türkiye the traditionalists, as well as some managers who otherwise favour modernisation, claim that too many university‐educated nurses attach no value to the messy aspects of the work and seek to substitute academic knowledge for experiential knowledge of how to care for real patients. The tensions between these orientations, with their differing visions of how the hospital should be managed, help explain the widespread negativity towards managers and the venom of the comments made in the interviews.

### 3.2 Pragmatists

Not all‐graduate entrants show commitment to professional development. A sizable group of ‘new nurses’ have a utilitarian, pragmatic orientation, attracted by the reasonable salary, job security and limited demands of the work. For this group nursing is a job rather than a vocation.

The new generation of nurses choose nursing primarily for the money and secondly because it is a profession where you easily get a post. Not because they are passionate or love nursing. As a result, the quality of the nursing profession is declining. They don't even give proper nursing care anymore. The idea of being a comfortable civil servant (…) is why people choose this profession.

(DM6‐F‐University Hospital‐BSN‐YE14)

‘New generation’ nurses came in for criticism from nurses adopting both traditionalist and moderniser standpoints. The former argued that new nurses showed the same lack of close engagement with patients as over‐educated professional modernisers, and that notwithstanding their lowly status still considered many nursing tasks to be below them.

New generation nurses are very different. (…) They do not want to touch the patient and they do not want to take any responsibility at all.

(M4‐F‐ City Hospital‐PhD‐YE17)

Modernisers complain about the poor interpersonal skills and crass communication style of many younger graduate nurses. They are said to get away with bad behaviour because civil service status means that they are unlikely to be disciplined.

New nurses are very defensive and abrupt which makes communication difficult. If they were not civil servants they wouldn't do these things and it would be easier to talk. We do not have audit or control at all. Among nurses, the quality of communication needs to improve in terms of tone of voice, calling colleagues by their name, that kind of thing.

(DM5‐F‐University Hospital‐BSN‐YE16)

The theme coming through in several nurse managers' accounts is that new nurses take advantage of the protections that civil service status provides, while not complying with other aspects of the old officer culture. In particular, they do not pay sufficient attention to hierarchy or give superiors the respect they expect.

New generation nurses do not care about hierarchy. They go to their managers and top managers directly. New generation nurses do not show respect. (…) I find difficult to communicate with new nurses, they know nothing, they cannot even give an injection. But they talk a lot, while they do not listen at all.

(M5‐F‐MoH‐Hospital‐MSN‐YE16).

Newly graduated nurses are very arrogant and they think they need to be managers. They finish school and get a master's degree in management right away, and they wonder ‘how can someone else can be a manager while I'm around’.

(DM6‐F‐University Hospital‐BSN‐YE14)

Despite the large number of ‘new generation’ nurses in hospitals, none of our informants said they fell into this category or talked about their resistance or indifference to the professionalising discourse espoused by colleagues. This may reflect bias in our sample, which did not include nurses with less than 5 years' service. We suspect that pragmatists were less likely to be named when we asked experienced nurses recruited via the Executive Nursing Association to nominate colleagues to expand our snowball sample.

### 3.3 Bureaucratic Modernisers

The HTP incorporated many NPM‐style reforms, including elements of performance management, audit and incentive payments that were implemented to a greater or lesser extent in the healthcare system. Terms from NPM had found their way into the language of nursing management, but the degree of enthusiasm for NPM‐style management practices varied markedly among the managers in the study. Only a minority, such as the following respondent, described their practices in these terms.

Annual planning is done. We are involved in this. What do we need for the team? What training do we need to organise? We give the latest approval for this planning. We are also following up on this. Whether various types of training are carried out or not, what they contribute… We look at the results. Then I can oversee everything that can affect patient care. How is patient satisfaction measured? (…) We do auditing, together with nurses and health care technicians, of course.

(DM4‐F‐City Hospital‐PhD‐YE17)

Auditing nursing performance was mentioned by several respondents as a key management task, and this included some nurses whose expressed views otherwise aligned more closely to the traditionalist or professional moderniser orientations. In some interviews management identity and preparation for management work was foregrounded more than professional nursing identity.

When I go on ward visits, I need to be super‐knowledgeable and draw on my experience to observe and audit the staff. You need to know what you are doing. We had management lessons at university. I also studied for a healthcare management master's degree.

(M5‐F‐MoH Hospital‐MSN‐YE16)

Only a few nurse managers interviewed might be said to have crossed the Rubicon to see themselves more as managers than nurses. A more common pattern was to use some terms from the language of NPM when they described their roles, but at the same time highlight aspects of their work that related to professional nursing concerns such as quality standards, improvements in nursing care and patient experience.

My first responsibility is managing human resources, planning, organising all nursing care delivery, inspecting the nursing services, make sure all the nursing care services are right, appropriate, well‐timed, well‐planned in advance. All the healthcare services must meet the quality standards. I am responsible for controlling and improving these quality standards. I am responsible for all the services that are given to patients from admission to discharge. (…) I spend lots of time controlling and inspecting the quality standards, outcomes and applications. Discipline, audit, motivating the staff, controlling all the wards and patient rooms to make sure patients are getting high quality care. (…) We try to record everything digitally with less paper, so we work on what are the needs for nursing care, what kind of documents need to be on the system et cetera: right documentation, right way, and a practical way of doing it.

(M4‐F‐City Hospital‐PhD‐YE17)

### 3.4 Professional Modernisers

However, a bigger group of nurse managers preferred to emphasise nursing identity and the advancement of nursing's professional project. This professional project centres on developing a nurse leadership role that foregrounds continuing professional education, improved patient care, evidence‐based practice and developing nursing standards. The professional moderniser orientation advances its agenda by means such as moving more nurses with higher degrees into nurse management positions, and building teamwork, although only rarely interdisciplinary teamwork. Where possible, modernisers seek to build good relations with sympathetic doctors, but where this is too difficult, they create spaces to exercise autonomy in areas where the nursing domain can be kept separate from doctor interference.

If you work in a hospital where the educational level of nurses is high, physicians look at you from within that framework. In other words, if the nurse's level of education is high, the nurse's ability to present herself well is high, and if she gives quality care she respects herself professionally. If you have autonomy, the physician respects you, and you have good communication. (…) Nurses are a very mixed group. If a nurse brings coffee for the physician or prepares breakfast, then that creates a profile where the nurse does what the physician says. Then the physician, of course, makes conversation according to that situation. This causes us to be unprofessional. It shakes our professional image and affects the professional satisfaction of nurses.

(DM2‐F‐University Hospital‐PhD‐YE20)

Evidence‐based practice was mentioned as an important component of professional development by several respondents, and most hospitals have quality units working in this area. However, developing, and more crucially implementing guidelines in routine ward practice is difficult because this usually depends both on medical sponsorship and support from nurse managers who may have a traditional mindset.

Lack of knowledge is the problem. (…) there are those who confuse nursing diagnosis with medical diagnosis. (…) There is a lot of resistance to change in public hospitals, and the people don't want to learn anything or do anything new. There is resistance to change and innovation. Since public hospitals are traditional they carry the logic that they should remain as they are, not learn anything new. But when I was working in a private hospital, it was the opposite. (…) I knew the manager, she was open to new ideas, at least he was like that with me. She valued my ideas because I was doing my PhD. They were very hungry for innovation. but there is absolutely no such mentality in public hospitals ‐ traditionalist and traditional. (…) I already had a scale that measured innovation and innovative behaviours. (…) Nurses get stuck on obstacles the most. (…) They can't turn innovation into innovative behaviour. They can't turn it into a service.

(N17‐M‐University Hospital‐BSN‐YE6)

The importance of interpersonal interaction in clinical work and the nurse leader's ability to create an environment that encourages harmonious staff relations was also mentioned by several respondents.

I was told that my strengths ‐ communication and the empathy side ‐ are very strong. Also, analytical thinking, problem solving, planning, organising, communication skills. They say I'm the transformational leader. Being patient and a good communicator are important. I've had a lot of education and training. I tried to continuously improve myself. I have a master's degree; I didn't want to do a PhD because our education here is just theory. I've also had process management training, project management training, leadership training, Lean training, Six Sigma training, quality training.

(M3‐F‐University Hospital‐BSN‐YE21)

Although the interviews included a question about the form of leadership that participants favoured, this was one of just two interviews mentioning a specific leadership style described in the Western nurse leadership literature (Cummings et al. 2018; Specchia et al. 2021). Only transformational leadership was mentioned, although other respondents described an approach resembling a relational leadership style, including elements such as close engagement with subordinates, collaboration, good communication and mutual influence. Given that participants had so little to say about the Western nurse leadership literature it would be premature for us to speculate about just how far the perspectives of Turkish nurses align with or diverge with key ideas in current debates. This is an intriguing area that might be explored in future studies.

In Türkiye the transmission of ideas about nursing modernisation comes less from direct contact with overseas nurses than via communication channels such as professional journals and conferences. Few non‐Turkish nationals are employed as nurses in Turkish hospitals, and no nurse in the sample had qualified in a Western country.

### 3.5 A Space for Nursing Autonomy?

*Professional modernisers* believed that nursing practice development was being held back, not just by medical dominance and the civil service culture, but by colleagues who did not share their vision. They realised that doctor power and the influence of the ‘officer culture’ are unlikely to change without further major healthcare reforms that are outside their control. Instead, the *professional modernisers* mostly concentrated on bottom‐up initiatives that they hoped will engender cultural change through education, team building, nursing guideline development and slowly modifying work practices and patterns of communication. Their aim is to transform nursing from within, rather than to gain greater decision‐making authority at the higher levels of management controlled by doctors. Their professionalising project is therefore a deliberately modest and restrained one, which seeks to change what nurses do in their own work domain, while avoiding a direct challenge to medical authority. Hence there is no push to develop hybrid roles or to gain seats in key management committees that determine hospital policies. As we explain elsewhere (Inceoz and Hughes 2026), what emerges is a curtailed form of nursing autonomy found mainly in certain hospital locales where nurses operate under less intensive medical supervision. This tends to centre on specialist wards, emergency units or as in the following interview extract, intensive care units, where nurses engage in shared decision‐making in teams in which they have greater than usual influence.

We have a very good team relationship and good communication. (…) Whether the patient is in a bed for one day or forty days, every detail is shared over and over again from the very beginning between doctors and nurses. (…) They evaluate the patient together. Our communication is high quality and very useful indeed. We attend bedside visits, share detailed information with the physician about the patient, and evaluate them together. No detail is skipped, everything is noted and shared. Whatever is needed is done immediately. (…) A care treatment plan is made. We will definitely exchange ideas.

(C5‐F‐MoH Hospital‐MSN‐YE10)

Such locales are akin to separate socio‐cultural enclaves within the larger hospital, typically having a distinctive atmosphere and ways of working that are controlled by senior nurses with many years' experience in the specialty. Compared with the general wards, specialist units typically have a more stable staff group, fewer staff passing through on short‐term rotation and less intensive oversight by nursing and medical managers.

In some cases, nurses were able to form teams that were predominantly nursing teams, subject to only limited medical ‘interference’.

We are on a track where doctors and nurses cannot be a team, but we [nurses] work well as a team in our own wards. (…) As a team we support each other a lot. We have good communication between us. (…) The head physician does not interfere in the affairs of the nurses. (…) None of the nurses in our department say, ‘this is not my patient, I cannot take the IV fluid, I won't take care of your patient et cetera’. We help each other, teamwork is very strong among us.

(C1‐F‐MoH Hospital‐BSN‐YE26)

In such cocoon‐like environments, nurses are more able to implement their own ideas about effective ways of working. Respondents who described these teams communicated a strong sense of in‐group identity, in several cases buttressed by WhatsApp or email groups and shared social activities.

## 4 Discussion

The four groups described above are ideal types—analytical constructs intended as theoretical reference points rather than mutually exclusive categories into which participants neatly fall. As in other studies of professional segmentation, nurses often expressed inconsistent or even contradictory views. Only a small minority demonstrated a strong commitment to NPM, yet many more spoke favourably about specific NPM mechanisms such as nursing audit. At the same time, several respondents who endorsed the language of nursing modernisation and service innovation nevertheless believed that a hospital environment shaped by medical dominance and a civil‐service ethos was unlikely to change in the foreseeable future. This perception fostered a pragmatic acceptance of patronage and dependence on sympathetic superiors, even among those who rhetorically supported reform.

Although our fourfold typology emerged inductively from the data rather than from prior literature, it resonates with earlier analyses of professional segmentation. In particular, it echoes the three‐way division between professionalisers, traditionalisers and utilisers identified in Habenstein and Christ's (1955) classic study of American nurses. Their ‘utiliser’—a nurse who approaches the occupation instrumentally, treating it primarily as a job—finds parallels in both the traditionalists and the self‐serving pragmatists in present‐day Türkiye. However, civil‐service employment (absent in the US context) gives traditionalism a distinctive form, embedding it within a bureaucratic culture of stability and hierarchy. Moreover, the rise of neoliberal policy frameworks since the late 20th century has introduced new reform discourses into Turkish nursing, producing two distinct modernising orientations, only one of which aligns with the classic ‘professionaliser’ category.

Subsequent studies reveal both continuity and transformation in these patterns. Leininger (1970) identified two subcultures among U.S. nurses in the late 1960s—an ‘old’ and a ‘new’ culture—closely resembling the traditionalisers and professionalisers. The traditional culture was marked by passivity, other‐directedness, self‐sacrifice and deference to paternalistic figures such as physicians and administrators. The new culture, by contrast, emphasised shared decision‐making with physicians, independence in practice, pursuit of nurses' own goals and the use of collective bargaining to secure improved pay and working conditions.

By the 1970s, the rational, modernising orientation had begun to branch in multiple directions. Carpenter's (1977) study of British nursing identified three groups: new managers, new professionals and the rank and file. The new managers embodied a bureaucratic line‐management model emerging from the Salmon Report reforms, while the new professionals were clinical specialists whose work overlapped with junior doctors. The rank and file represented the mainstream, accepting nursing as largely doctor‐devolved work. Melia (1987) later added a fourth group—the academic professionalisers—whose work was partly university‐based and involved less direct patient care. These broad patterns persisted in the UK into the late 20th century (Dent and Burtney 1997), with more recent developments centring on interdisciplinary working, distributed leadership and hybrid roles.

Following Bucher and Strauss's (1961) classic formulation, we argue that professional segmentation is a recurrent feature of professional development, emerging and evolving over time. Segmentation reflects both a profession's response to endogenous changes—such as health reforms that elicit divergent reactions from different groups—and internal deliberations shaped by transnational discourses of professional development. Modernising segments such as professionalisers and NPM‐style managers can be understood as initiators of occupational ‘social movements’ seeking to reshape the profession in preferred directions (Coe 1970). As these segments interact and compete, they construct representations of themselves and others, and of the legitimacy and feasibility of their respective occupational visions. This dynamic is evident in our interview data, where accounts are often coloured by rhetoric, exaggeration and what Allen (2001, 76) terms ‘atrocity stories’—dramatic or shocking narratives that acquire legendary status within occupational cultures.

Overall, the segmentation observed in Turkish nursing bears a family resemblance to earlier patterns described in the literature, but with important differences shaped by socio‐cultural and institutional context. Civil‐service employment gives traditionalism a bureaucratic inflection; entrenched medical dominance constrains the scope of professionalisers' ambitions, and neoliberal reforms have generated a second modernising orientation—bureaucratic modernisation—that is distinct from professionalisation. Interest in audit, performance management and quality assurance is visible among a minority of bureaucratic modernisers, but they remain outnumbered by nurse managers who continue to favour a professional identity.

## 5 Limitations

This paper presents a case study of nurse management in public hospitals in Istanbul, a city in the European region of Türkiye where institutional cultures and exposure to professional modernisation may differ significantly from the situation in rural, conservative and less well‐resourced regions. While our 40 interviews provide multiple cross‐cutting accounts of management practices in five hospitals, the study lacks the scale to demonstrate with confidence any associations between the four orientations to nursing work described and the demographic characteristics of the participants. We must also acknowledge that our recruitment strategy and inclusion criteria limited the number of recently qualified nurses and older nurses who entered the profession by a non‐degree route, so that few participants in our sample represent the traditionalist or pragmatist orientations. Our qualitative study provides a rare and valuable insight into nurse management in one large Turkish city, but should ideally be complemented by larger cross‐sectional surveys that will help assess the generalisability of the findings.

## 6 Conclusions

The future trajectory of nursing modernisation in Türkiye remains uncertain. The increasing number of graduate nurses entering management, combined with the impending retirement of traditionalists, suggests that the professionalising project will gain momentum. Yet an alternative scenario is also plausible: a future weakening of medical dominance could shift nurses' primary identity towards executive leadership rather than professional expertise. Although there is little evidence of such a shift at present, future developments may produce new tensions between professionalisers and bureaucratic modernisers as they compete to shape the direction of nursing management. Whether Turkish nursing will follow the trajectory observed in the United Kingdom and other Western countries—where managerial identities have become increasingly prominent—or pursue a distinct professional pathway also followed by other middle‐income countries remains an open and important question.

## AI Usage

AI (Co‐pilot) was used for background literature searches, but not in the analysis of data.

## Author Contributions

**Zuleyha Inceoz:** conceptualization (equal), investigation (lead/sole), methodology (equal), formal analysis (lead), writing – original draft (equal), writing – review and editing (equal). **David Hughes:** supervision (lead), conceptualization (equal), methodology (equal), formal analysis (supporting), writing – original draft (equal), writing – review and editing (equal).

## Funding

The authors have nothing to report.

## Ethics Statement

Ethical approval for this study was granted by the Research Ethics Committee of the Faculty of Medicine, Health & Life Science, Swansea University, UK, on 12 February 2021 (reference number 130121), and by the Research Ethics Committee, Mamara University, Istanbul, Türkiye on 22 February 2021 (reference number: 20‐2‐21‐13). All respondents gave informed consent in writing to participate and to allow publication of results prior to the start of the study.

## Conflicts of Interest

The authors declare no conflicts of interest.

## Data Availability

The data used in the study comprised recordings of Zoom interviews, interview transcripts in the Turkish language and English language translations of selected passages. Following analysis, the Zoom recordings were deleted, and the authors have decided not to make full or partial interview transcripts available for reasons of preserving the anonymity of participants and also the complications of translation.

## Footnotes

1 This paper is based on Zuleyha Inceoz's PhD research (Inceoz 2024), which provides a more detailed account of Turkish healthcare reform, the study methodology, a review of relevant literature and reflections on the Turkish political context and the forces likely to shape or limit future reform of nursing management. The thesis contains more extensive extracts from the interviews.

^2^
Interview extracts are labelled as follows. The first letter indicates nurse grade: senior nurse manager (M), supervisor (S), deputy manager (DM) charge nurse (C) and staff nurse (N). The number that follows identifies the respondent. M or F refers to male or female gender. Next, the type of hospital is indicated, followed by degree (Bachelor's, Master's or PhD). Lastly, number of years of experience (YE) in nursing is specified. Supervisors oversee the hospital at night.
